# SUMOylating Two Distinct Sites on the A-type Potassium Channel, Kv4.2, Increases Surface Expression and Decreases Current Amplitude

**DOI:** 10.3389/fnmol.2019.00144

**Published:** 2019-05-31

**Authors:** Meghyn A. Welch, Lori A. Forster, Selin I. Atlas, Deborah J. Baro

**Affiliations:** ^1^Department of Biology, Georgia State University, Atlanta, GA, United States; ^2^Neuroscience Institute, Georgia State University, Atlanta, GA, United States

**Keywords:** SUMOylation, SUMO, Kv4, ion channel, trafficking, voltage-gated potassium channel, A-type potassium current

## Abstract

Post-translational conjugation of Small Ubiquitin-like Modifier (SUMO) peptides to lysine (K) residues on target proteins alters their interactions. SUMOylation of a target protein can either promote its interaction with other proteins that possess SUMO binding domains, or it can prevent target protein interactions that normally occur in the absence of SUMOylation. One subclass of voltage-gated potassium channels that mediates an A-type current, I_A_, exists as a ternary complex comprising Kv4 pore-forming subunits, Kv channel interacting proteins (KChIP) and transmembrane dipeptidyl peptidase like proteins (DPPL). SUMOylation could potentially regulate intra- and/or intermolecular interactions within the complex. This study began to test this hypothesis and showed that Kv4.2 channels were SUMOylated in the rat brain and in human embryonic kidney (HEK) cells expressing a GFP-tagged mouse Kv4.2 channel (Kv4.2g). Prediction software identified two putative SUMOylation sites in the Kv4.2 C-terminus at K437 and K579. These sites were conserved across mouse, rat, and human Kv4.2 channels and across mouse Kv4 isoforms. Increasing Kv4.2g SUMOylation at each site by ~30% produced a significant ~22%–50% decrease in I_A_ G_max_, and a ~70%–95% increase in channel surface expression. Site-directed mutagenesis of Kv4.2g showed that K437 SUMOylation regulated channel surface expression, while K579 SUMOylation controlled I_A_ G_max_. The K579R mutation mimicked and occluded the SUMOylation-mediated decrease in I_A_ G_max_, suggesting that SUMOylation at K579 blocked an intra- or inter-protein interaction involving K579. The K437R mutation did not obviously alter channel surface expression or biophysical properties, but it did block the SUMOylation-mediated increase in channel surface expression. Interestingly, enhancing K437 SUMOylation in the K579R mutant roughly doubled channel surface expression, but produced no change in I_A_ G_max_, suggesting that the newly inserted channels were electrically silent. This is the first report that Kv4.2 channels are SUMOylated and that SUMOylation can independently regulate Kv4.2 surface expression and I_A_ G_max_ in opposing directions. The next step will be to determine if/how SUMOylation affects Kv4 interactions within the ternary complex.

## Introduction

The transient A-type potassium current (I_A_) is a rapidly activating, subthreshold current that regulates neuronal excitability. In mammals, I_A_ is mediated by Kv1.4, Kv3.3-4 and Kv4.1-3 α-subunits (Gutman et al., 2005). Kv4 channels are widely expressed in a number of central neurons, and evidence suggests that native Kv4 channels exist in a ternary complex with two well studied auxiliary subunit proteins, cytosolic Kv channel interacting protein (KChIP) and transmembrane dipeptidyl peptidase like protein (DPPL; Birnbaum et al., 2004; Jerng and Pfaffinger, 2014; Wang et al., 2015). Interactions between Kv4α subunits and auxiliary subunits determine channel surface expression and biophysical properties (An et al., 2000; Holmqvist et al., 2002; Nadal et al., 2003; Shibata et al., 2003; Jerng et al., 2004; Zagha et al., 2005; Jerng and Pfaffinger, 2014). However, how these interactions are regulated remains largely unknown. KChIP EF-hands can bind Mg^2+^ and Ca^2+^, and there is evidence suggesting Ca^2+^ binding to KChIP can influence its interaction with Kv4α (Morohashi et al., 2002; Chen et al., 2006; Pioletti et al., 2006; Lee et al., 2009; Gonzalez et al., 2014; Bähring, 2018). N-glycosylation of DPP10 controls its interaction with Kv4α, and its ability to modulate channel surface expression and biophysical properties (Cotella et al., 2010, 2012). It is important to identify additional mechanisms that may govern these interactions. Post-translational modifications like SUMOylation can regulate protein-protein interactions, and in this work, we begin investigating whether SUMO regulates subunit interactions by studying the role of Kv4 SUMOylation.

Small ubiquitin-like modifier (SUMO), a.k.a. sentrin, represents a family of pro-peptides. There are four SUMO isoforms in mammals (SUMO 1-4). SUMO-1 and SUMO-2 share 47% sequence identity. SUMO-2 and SUMO-3 are 97% identical and are typically referred to as SUMO-2/3 because they are usually not experimentally differentiated. Post-translational modifications by SUMO 1-3 are well studied (Flotho and Melchior, 2013; Wasik and Filipek, 2014; Henley et al., 2018). SUMO-4 is atypical and little is known about its function as a post-translational modifier (Guo et al., 2004; Owerbach et al., 2005; Wei et al., 2008; Wang et al., 2009).

The terminal amino acids of SUMO 1-3 pro-peptides are removed to produce a mature peptide that ends in a di-glycine. The mature peptide can be conjugated to lysine (K) residues on target proteins by the enzyme, Ubc9 (Desterro et al., 1997). In most cases (~65%), Ubc9 recognizes a SUMO consensus sequence on the target protein and SUMOylates the K in that sequence; however, Ubc9 can also act in conjunction with other proteins to SUMOylate K residues at non-consensus sites (Matic et al., 2010; Flotho and Melchior, 2013; Hendriks et al., 2015). SUMO modification is reversible, and deconjugation is effected by a family of sentrin-specific proteases (SENP; Hickey et al., 2012).

SUMO influences diverse cellular functions by controlling protein-protein interactions, and recent work highlights SUMO’s role in regulating the surface expression and biophysical properties of ion channels (Kruse et al., 2009; Wasik and Filipek, 2014; Gong et al., 2016; Wu et al., 2016; Parker et al., 2017; Henley et al., 2018). Several voltage-gated potassium channels are known to be regulated by SUMO. Kv1.1 channels are modified by SUMO-1 and SUMO-2/3 and co-localize with SENP2 in hippocampal neurons (Qi et al., 2014). Kv1.5 channels are largely SUMOylated at two K residues, and preventing SUMOylation at these sites causes a hyperpolarizing shift in the voltage of half-inactivation (V_50_ inact; Benson et al., 2007). SUMOylation of Kv2.1 channels regulates pancreatic β-cell excitability by accelerating the time constant (τ) of inactivation and impairing recovery from inactivation (Dai et al., 2009). Additionally, Kv2.1 SUMOylation increases the excitability of rat hippocampal neurons by causing a depolarizing shift in the voltage of half-activation (V_50_ act; Plant et al., 2011). Kv7.1 channel SUMOylation in neonatal mouse ventricular myocytes results in a depolarizing shift in V_50_ act (Xiong et al., 2017). SENP2 knockout mice display increased excitability in CA3 pyramidal neurons due to hyper-SUMOylation of Kv7.2 channels which diminishes the hyperpolarizing M-current, causing these mice to develop spontaneous convulsive seizures followed by sudden death (Qi et al., 2014). Kv11.1 SUMOylation decreases steady-state current amplitude by decreasing the τ of inactivation and modifying deactivation kinetics (Steffensen et al., 2018).

SUMOylation of Kv4 channels has not been reported. Here, we test the hypothesis that Kv4.2 channels are SUMOylated to regulate their surface expression and biophysical properties.

## Materials and Methods

### Plasmids and Antibodies

A previously described plasmid containing a mouse Kv4.2-GFP fusion protein, here termed Kv4.2g, was generously provided by Dr Dax Hoffman. Note GFP is attached to the C-terminus of Kv4.2. A plasmid containing mCherry2-Cl was a gift from Michael Davidson (Addgene plasmid #54563). A plasmid containing SUMO-2 (Kamitani et al., 1998) was a gift from Edward Yeh (Addgene plasmid #17360). A plasmid containing Ubc9 (Yasugi and Howley, 1996) was a gift from Peter Howley (Addgene plasmid #14438). All antibodies are described in Table 1.

**Table 1:** Primary antibodies.

Antigen	Verified by	Species, manufacturer and catalog number	Concentration used
Kv4.2	Specificity verified by the company. WB analysis of rat membranes probed with anti-Kv4.2 (1:200) revealed a 68 kD band.	rabbit, alomone labs, #APC-023	IP: 8.3 μL antibody per 1,000 μg protein; WB: 1:750
GFP (IP)	Specificity verified by the company. Ab detects 5 ng recombinant GFP expressed in HEK293 cells.	rabbit, abcam, ab290	IP: 1 μL antibody per 500 μg protein
GFP (WB)	Specificity verified by the company. WB analysis of untransfected COS and COS cells transfected with pCruz GFP-Lac Z	rabbit, Santa Cruz Biotechnology, sc-8334	WB: 1:3,000
SUMO-1	Specificity verified by the company. WB analysis of HEK293 expressing SUMO-1.	rabbit, Santa Cruz Biotechnology, sc-9060	WB: 1:1,000
SUMO-2/3	Specificity verified by the company. WB analysis of HEK293 cells transfected with SUMO-2	rabbit, Santa Cruz Biotechnology, sc-32873	WB (HEK cells): 1:3,000; WB (rat brain): 1:1,000
SUMO-2/3	Specificity verified by the company. Ab recognizes 15 and 18 kD bands in HeLa extract on WB.	rabbit, abcam, ab3742	WB (HEK cells): 1:1,000; WB (rat brain): 1:750
Na^+^/K^+^-ATPase	Specificity verified by the company. Positive signal on WB using HEK293 whole cell lysate.	mouse, abcam, ab7671	WB: 1:3,000
Actin	Specificity verified by the company. WB analysis of C32 whole cell lysate	rabbit, Santa Cruz Biotechnology, sc-1616-R	WB: 1:2,000
BSA	Specificity verified by the company. BSA was loaded onto a gel. WB analysis revealed a 68 kD band when probed with anti-BSA antibody.	rabbit, ThermoFisher, A11133	WB: 1:20,000

### Site-Directed Mutagenesis

PCR-based site-directed mutagenesis was used to create two mutations in the Kv4.2g plasmid described above: K437R and K579R. Typically, 10–100 ng of Kv4.2g DNA served as the template in 50 μL PCRs containing PrimeSTAR GXL polymerase (Takara), 5X PrimeSTAR GXL buffer (Takara), nucleotides, and the primers described in Table 2. The cycling conditions were as follows: 1× 95°C 1 min; 30× 98°C 30 s, 68°C 7 min; 1× 68°C 5 min. Afterward, 20 units of DPN1 (Takara) was added to the PCR and incubated at 37°C for 2 h to digest the template DNA. Then, 2 μl of the PCR was added to 50 μl of subcloning grade competent XL1 blue cells (Agilent) and incubated on ice for 30 min. The cells were heat shocked for 45 s at 42°C and cooled on ice for 2 min. Two-hundred microliter of NZY both was added and the reaction was incubated at 37°C for 30 min to allow for the expression of kanamycin resistance. The cells were plated onto NZY plates containing 30 μg/ml kanamycin and incubated at 37°C overnight. A single colony was used to inoculate NZY broth containing 30 μg/ml kanamycin and incubated at 37°C, shaking at 220 rpm, overnight. Plasmid DNA was isolated using the Qiagen Mini Kit, according to the manufacturer’s instructions. The isolated plasmid was sequenced at the Georgia State University Cell Protein and DNA Core Facilities, and the sequences were analyzed using Lasergene software (DNAstar) to ensure that only the desired mutation was present. To create a double mutation, a previously mutated Kv4.2g channel served as the template in the PCR.

**Table 2:** Site-directed mutagenesis primers.

Primer name	Primer sequence
For-Kv4.2K437R	CGAGCAGCCAgAAGCGGGAGTGCAAATGCCTACATGC
Rev-Kv4.2K437R	GCATGTAGGCATTTGCACTCCCGCTTcTGGCTGCTCG
For-Kv4.2K579R	CAGCCGATCCAGCTTAAATGCCAgAATGGAAGAGTGTGTTAAAC
Rev-Kv4.2K579R	GTTTAACACACTCTTCCATTcTGGCATTTAAGCTGGATCGGCTG

### Rat Brain Membrane Preparations

A single whole rat brain was homogenized ~20× on ice in homogenization buffer [0.3M sucrose, 10 mM sodium phosphate buffer pH 7.4, 1 mM EDTA, and protease inhibitor cocktail (1:100, Sigma cat. #P8340)] supplemented with 20 mM *N*-Ethylmaleimide (NEM) to prevent SUMO deconjugation. The homogenate was centrifuged at 5,000 rpm for 10 min at 4°C to spin down cell nuclei. The supernatant was transferred to two Beckman tubes and crude membranes were pelleted by centrifuging at 40,000 rpm for 90 min at 4°C. The supernatant was removed, the pellets were re-suspended in homogenization buffer (500 μl/tube) for 1 h at 4°C with shaking. The resuspended pellets from each tube were combined, and protein concentration was determined by performing a bicinchoninic acid (BCA) assay (Pierce). Rat brain tissue was generously provided by Dr Chun Jiang. This study was carried out in accordance with the principles of the Basel Declaration and recommendations of Ethical Issues of the International Association for the Study of Pain and National Institutes of Health. The protocol was approved by the Institutional Animal Care and Use Committee at Georgia State University.

### Cell Culture

Human embryonic kidney 293 (HEK-293) cells were obtained from American Type Culture Collection (ATCC). Cells were maintained at 37°C with 5% CO_2_ in Eagle’s Minimum Essential Medium (Corning, cat. #10009CV) supplemented with 10% fetal bovine serum (FBS; ATCC 30-2020) and 1% Penicillin/Streptomycin (Sigma, cat. #P4333).

### Generating Cell Lines Stably Expressing Wild-Type and Mutant Kv4.2g

HEK-293 cells were plated on 60 mm culture plates at ~90% confluency. One hour prior to transfection, the media was replaced with EMEM+10% FBS without antibiotics. Ten microgram of plasmid DNA was combined with 50 μl OptiMEM (Gibco). Twenty microliter of Lipofectamine 2,000 (Invitrogen) was combined with 50 μl OptiMEM. After 5 min, DNA and Lipofectamine were combined and incubated for 20 min at RT. The mixture was dropped onto HEK-293 cells. After 2 days, cells were harvested and dilute resuspensions were re-plated with EMEM+ 10% FBS+ 1% Pen/Strep+ G418 (Geneticin, Gibco, 500 μg/ml). After ~3 weeks individual colonies were selected using cloning rings. Expression in all cells was confirmed using fluorescence microscopy and whole cell patch clamp recordings.

### Transient Transfections

Cells were plated onto 60 or 100 mm culture dishes at 1.9 × 10^6^ or 6 × 10^6^ cells per plate, respectively. The next day, cells were transiently transfected using calcium phosphate. At least 1 h prior to transfection, the media was changed. For 60 or 100 mm culture plates, 10 or 25 μg of plasmid DNA was prepared in 250 or 440 μl of TE buffer (10 mM Tris-HCl pH 8, 1 mM EDTA), respectively. When multiple plasmids were co-transfected, the total amount of DNA remained the same, and the different plasmids were equally represented by weight within a mixture. Immediately prior to transfection, 30 or 60 μl of 2M CaCl_2_ was added to the DNA, dropwise, flicking to mix. Then 250 or 500 μl of 2× HBS (275 mM NaCl, 10 mM KCl, 12 mM dextrose, 1.4 mM Na_2_HPO_4_, 40 mM HEPES, pH 7.05–7.1) was added dropwise to the DNA, flicking to mix. The mixture was immediately added to the cells and cells were returned to 37°C with 5% CO_2_ for 4 h followed by a media change. Cells were allowed to grow for ~48 h to allow for the expression of the plasmid DNA. Before using the transfected cells, the transfection efficiency was assessed using fluorescence microscopy. Only plates with >80% transfection efficiency were used in experiments.

### Immunoprecipitation

#### Kv4.2 IP From Rat Brain

Kv4.2 channels were immunoprecipitated from rat brain membranes using the Classic Magnetic IP/Co-IP Kit (Pierce), 1 mg of rat brain membrane and 5 μg of rabbit anti-Kv4.2 (Table 1). The IP product was eluted in a volume of 50 μL.

#### GFP IP From HEK Cells

Cells on 100 mm culture dishes were washed 2× with ice-cold PBS and lysed with 1 ml of RIPA buffer (1% NP40, 50 mM Tris-HCl pH 7.4, 150 mM NaCl, 0.1% SDS, 0.5% DOC, 2 mM EDTA, 20 mM NEM, 1:100 protease inhibitor cocktail) for 30 min on ice. The plate was scraped, the lysate was transferred to a 1.5 ml microcentrifuge tube, and cell debris was spun down by centrifuging for 15 min at 14,000 rpm. Protein concentration in the supernatant was determined with a BCA assay (Pierce). Kv4.2g channels were immunoprecipitated using the Classic Magnetic IP/Co-IP Kit (Pierce) according to the manufacturer’s instructions using 5 μg of rabbit anti-GFP (Table 1) with 1 mg of protein. IP product was eluted in a volume of 100 μL.

### Western Blot

After electrophoresis on 12% SDS-polyacrylamide gels, proteins were transferred for 2 h at 45 mAMP to a PVDF membrane (Immobilon-P, cat. #IPVH00010) using a semi-dry electroblotting system (OWL). After drying overnight, membranes were blocked in 5% non-fat dry milk in TBS (50 mM Tris-HCl pH 7.4, 150 mM NaCl) for 1 h at room temperature. Blots were washed 1× for 10 min with TTBS (TBS+ 0.1% Tween20), and then primary antibodies prepared in 1% non-fat dry milk in TTBS were added and incubated at 4°C overnight with shaking. Blots were washed 3×, 5 min each with TTBS and then incubated with appropriate alkaline phosphatase conjugated secondary antibodies in TTBS with 1% non-fat dry milk for 2 h at RT with shaking. The membrane was washed 3× with TTBS, 10 min each. The blot was incubated with alkaline phosphatase substrate (Bio-Rad) for 5 min, and then the membrane was exposed to film (MedSupply Partners), and chemiluminescent signals were captured with a Kodak X-Omat 2000A imager. Optical densities for bands of interest were measured with ImageJ, as previously described (Parker et al., 2017). In some cases, antibodies were stripped from blots as follows: 2× mild stripping buffer, 10 min each; 2× PBS, 10 min each; 2× TTBS, 5 min each. AP substrate was applied for 5 min, and the membrane was exposed to film to ensure that the original chemiluminescent signal was gone. In order to compensate for error introduced by technical variabilities such as fluctuating exposure times and loss of protein due to stripping, blots that were stripped and re-probed always contained 0.2 μg BSA in one lane. A primary antibody against BSA was always included along with other primary antibodies to detect the BSA signal. The pre- and post-stripping BSA signals were used to normalize other signals of interest on the pre- and post-stripped blot, respectively.

### Biotinylation Assays to Measure Surface Expression

Cells were washed twice with room-temperature PBS containing 0.2 mM CaCl_2_ and 1.5 mM MgCl_2_ (PBS-CM) and incubated with 2 ml of 1 mg/ml EZ-link-Sulfo-NHS-SS-Biotin (ThermoFisher, cat. #21331) for 30 min at 8°C. Cells were washed twice with room-temperature PBS-CM and residual biotin was quenched using PBS-CM with glycine for 15 min at 8°C. Cells were lysed for 30 min on ice using 500 μL RIPA buffer. Cells were scraped from the plate and the lysate was transferred to a sterile 1.5 ml microcentrifuge tube. Cell debris was pelleted by centrifuging for 10 min at 14,000 rpm. The supernatant was removed, added to a spin column (ThermoFisher, cat. #69725) containing 100 μL of NeutrAvidin Agarose Resin (ThermoFisher, cat. #29201), and incubated with shaking for 2 h at 4°C. The lysate/resin was centrifuged at 1,000× *g* for 2 min and the eluate containing intracellular proteins was saved for western blot analysis. The resin was washed 3× with Wash Buffer 1 (1% NP40, 1% SDS, 1× PBS) and 3× with Wash Buffer 2 (0.1% NP40, 0.5M NaCl, 1× PBS). In order to elute extracellular proteins, 50 μL of 1× SDS buffer (1× SDS, 0.1% Bromophenol blue, 100 mM DTT) was added to the beads and incubated with shaking for 1 h at room temperature. Beads were pelleted by centrifugation at 1,000× *g* for 2 min. The supernatant was recovered and used in western blot analyses.

Western blots containing intracellular and extracellular fractions as well as 0.2 μg BSA (~66 kD, Sigma cat. #A7517) were cut horizontally at the ~50 kD marker. Optical densities for bands on the upper portion of the blot were obtained using primary antibodies against GFP (Table 1) and BSA (Table 1). After obtaining the optical densities for the Kv4.2g and BSA bands, the upper portion of the blot was stripped and re-probed with primary antibodies against Na^+^/K^+^-ATPase (Table 1) and BSA. The Kv4.2g and Na^+^/K^+^-ATPase signals were each normalized by their respective BSA signal to remove error introduced by technical variabilities such as fluctuating exposure times and loss of protein due to stripping. Kv4.2g surface expression was then quantified by dividing the normalized Kv4.2g signal by the normalized Na^+^/K^+^-ATPase signal, which we previously showed did not change when SUMO availability was altered (Parker et al., 2017). In all experiments, the lower portion of the blot was probed with a primary antibody against actin to detect any intracellular contamination in the extracellular fractions. The experiment was excluded if actin was detected in the extracellular fraction.

#### Whole Cell Patch Clamp Electrophysiology

Glass coverslips were prepared by dipping in ethanol, air drying, and coating with 50 μg/ml Poly-L-Lysine for 1 h at 37°C. Poly-L-Lysine was removed, coverslips were washed 1× with dH_2_0, and allowed to air dry before use. Cells were transiently transfected with mCherry or mCherry+SUMO+Ubc9. Approximately 24 h after transfection, cells were seeded onto 20 mm Poly-L-Lysine coated coverslips at a density of 8 × 10^4^ cells per coverslip, and were incubated for ≥24 h before use. A coverslip was transferred to the recording chamber and continuously superfused with extracellular saline (in mM: 141 NaCl, 4.7 KCl, 1.2 MgCl_2_, 1.8 CaCl_2_, 10 glucose, 10 HEPES, pH 7.4, osmolarity ~300). Cells were visualized using an Olympus IX70 microscope and only cells expressing mCherry, visualized by red fluorescence, were patched. Fire polished borosilicate glass pipettes having a resistance between 2 and 5 MΩ were filled with intracellular saline (in mM: 140 KCl, 1 MgCl_2_, 1 CaCl_2_, 10 EGTA, 2 MgATP, 10 HEPES, pH 7.2, osmolarity ~290) and connected to a MultiClamp 700A amplifier (Axon Instruments). To generate a whole cell patch, a GΩ seal was formed by slight negative pressure. After forming a GΩ seal, gentle suction was used to rupture the membrane and only cells that maintained ≥700 MΩ seal after breaking through the membrane were used. Fast and slow capacitance transients and series resistance was compensated. To elicit I_A_, a 1 s −90 mV pre-pulse was followed by a 250 ms test-pulse from −50 mV to +50 mV in 10 mV increments. Offline subtraction of currents evoked following a pre-pulse to −30 mV was used to isolate I_A_. I_A_ G_max_ and voltage of half-activation (V_50_ act) were determined by converting peak current amplitude (I_peak_) to conductance (G) for each voltage step using the equation G = I_peak_/(V_m_−V_r_), where V_r_ = −86 mV, plotting G/G_max_ as a function of membrane potential, and fitting the resulting curve with a first-order Boltzmann equation. The steady-state inactivation properties of I_A_ were determined with a series of 1.4 s steps from −110 mV to −30 mV in 10 mV increments, each followed by a 200 ms test pulse to +20 mV. Steady-state voltage-dependence of half-inactivation (V_50_ inact) curves were generated by plotting I/I_max_ as a function of pre-pulse voltages and fitting the resulting curve with a first-order Boltzmann equation. To determine the fast (τ_f_) and slow (τ_s_) time-constants of inactivation a two-term exponential equation was used to fit the decay current elicited by 250 ms voltage-step to +50 mV following a pre-pulse to −90 mV.

#### Statistical Analyses

Data were analyzed using GraphPad PRISM 7 software. Each data set was assessed for normality and homogeneity of variance. Data were analyzed using parametric or non-parametric tests as indicated, including the student’s unpaired *t*-test, the Mann-Whitney *U* test, and the one-way analysis of variance (ANOVA) followed by a Tukey’s *post hoc* comparison when appropriate. In all cases, the significance threshold was set at *p* < 0.05. Data points >2 standard deviations from the mean were considered outliers and were excluded. In all cases, values reported represent the mean ± SEM.

## Results

### Kv4.2 Is SUMOylated in the Rodent Brain

**Figure 1:**
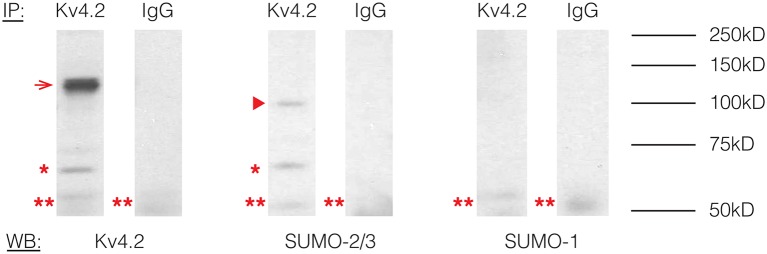
Kv4.2 channels are SUMOylated in the rodent CNS. Immunoprecipitation (IP) experiments were followed by western blot analysis. Antibodies are as indicated. The experiment was repeated three times using the brains from three different rats. A 68 kD band, representing the Kv4.2 channel is indicated by an asterisk. A 130 kD band representing aggregated Kv4.2 channels is indicated by an arrow. A 100 kD band that most likely represents a protein that non-covalently interacts with the Kv4.2 channel is indicated by an arrowhead. A 50 kD band (double asterisk) most likely represents the IP antibody.

To determine if Kv4.2 channels were SUMOylated *in vivo*, immunoprecipitation (IP) experiments were performed using rat brain membrane preparations and an antibody against Kv4.2 (Table 1) followed by western blot experiments using primary antibodies against Kv4.2, SUMO-1, or SUMO-2/3 (Table 1). The antibodies against Kv4.2 and SUMO-2/3, but not SUMO-1, recognized the same 68 kD band previously identified as the Kv4.2 channel in rat brain membrane preparations (Figure 1, asterisk; Nadal et al., 2003). This band was not observed when IP experiments were repeated with a non-specific rabbit IgG. These data suggest that a fraction of rat brain Kv4.2 channels was decorated with SUMO-2/3 and that the addition of the 12 kD modification did not detectably alter the molecular weight of the channel under our PAGE conditions. A 130 kD band likely representing aggregated Kv4.2 channels was also observed (Figure 1, arrow; Jerng et al., 2004, 2005). The anti-SUMO-2/3 antibody recognized an additional 100 kD band that was not recognized by the channel antibody in the anti-Kv4.2 but not the anti-IgG IP product (Figure 1, arrow head), and this band may represent a SUMOylated protein(s) that non-covalently interacts with the channel. Two likely candidates are DPP6-S and DPP10 that have predicted molecular weights of ~115 kD and ~100 kD when glycosylated, respectively (Jerng et al., 2004). SUMO prediction software indicates both of these proteins have potential SUMOylation sites. In sum, these results showed that Kv4.2 channels were measurably decorated with SUMO-2/3 but not SUMO-1 in the rat brain.

### Kv4.2 Channels Are SUMOylated in a Heterologous Expression System

**Figure 2:**
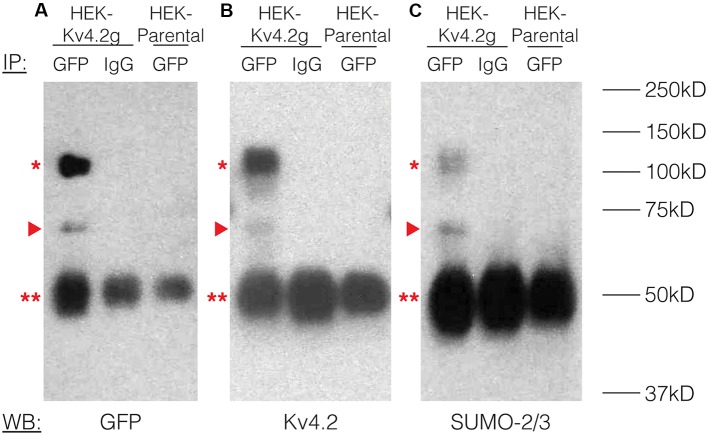
Kv4.2 channels are SUMOylated in a heterologous expression system. A mouse Kv4.2 channel with a C-terminal GFP tag (Kv4.2g) was stably expressed in Human Embryonic Kidney (HEK) cells (HEK-Kv4.2g). IP experiments were performed on cell lysates from HEK-Kv4.2g cells or HEK parental cells using an anti-GFP or a negative control IgG antibody. Western blots containing IP product were probed using an **(A)** anti-GFP, **(B)** anti-Kv4.2, or **(C)** anti-SUMO-2/3 antibody. Experiments were repeated three times. The Kv4.2-GFP protein was detected as a 100 kD band (asterisk) using antibodies against GFP, Kv4.2, and SUMO-2/3. This band was never observed when a negative control IgG or HEK parental cells were used in the IP. A 70 kD band was also detected by all antibodies (arrow head), and it most likely represents cleavage of the channel. This signal was not included in subsequent quantifications. This most likely represents sample degradation. The 50 kD band observed in all lanes (double asterisk) likely represents the IP antibodies.

To study the effects of Kv4.2 SUMOylation, we established a HEK-293 cell line that stably expressed Kv4.2g, which is a mouse Kv4.2 channel tagged with a C-terminal GFP. The stable cell line was called HEK-Kv4.2g. IP experiments using an anti-GFP antibody (Table 1) were performed on whole cell lysates from HEK-Kv4.2g cells, and IP products were analyzed with western blotting (Figure 2). Antibodies against GFP (Figure 2A), Kv4.2 (Figure 2B) and SUMO-2/3 (Figure 2C) all recognized the same 100 kD band (asterisk), which is the predicted size of the Kv4.2-GFP fusion protein. The 100 kD band was never observed when a non-specific IgG was used in IP experiments, or on blots containing IP products from HEK parental cells (Figure 2). Furthermore, a 70 kD band was also detected by all antibodies (arrow head), and it most likely represents cleavage of the channel. This signal was not included in subsequent quantifications, and most likely represents sample degradation. These data suggest that Kv4.2g channels are SUMOylated in our heterologous expression system under baseline conditions.

### SUMOylation of the Kv4.2 Channel Can be Manipulated in a Heterologous Expression System

**Figure 3:**
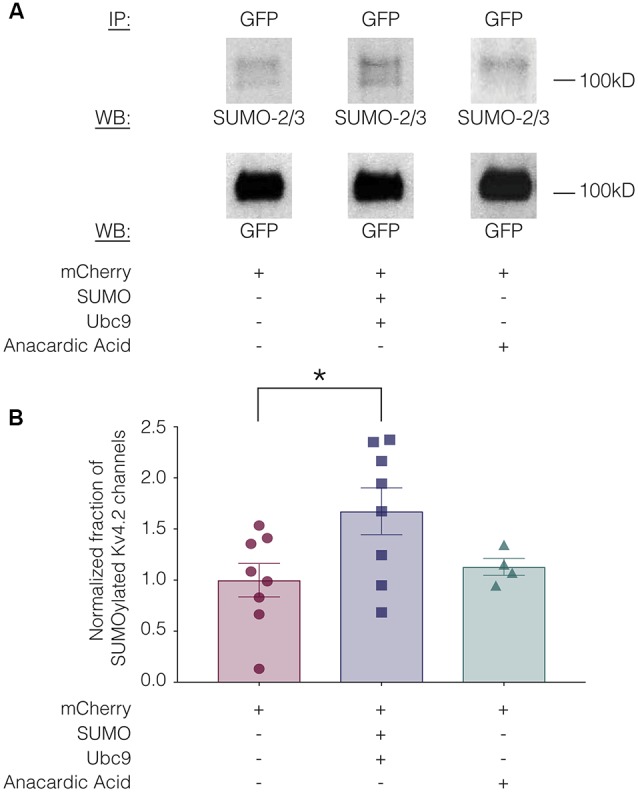
Kv4.2 channel SUMOylation can be manipulated in a heterologous expression system. HEK-Kv4.2g cells were transiently transfected with mCherry or mCherry+SUMO+Ubc9 plasmid DNA. Two days after transfection, cells were lysed and used in IP experiments. In some cases, HEK-Kv4.2g cells transiently transfected with mCherry were treated with anacardic acid (100 μM, 1 h) prior to cell lysis and IP. Western blots containing IP product were probed for SUMO-2/3 and then stripped and re-probed for GFP. The fraction of SUMOylated Kv4.2 channels was determined. **(A)** Representative western blots for each treatment group. Note that two bands are resolved with anti-SUMO-2/3, but not anti-GFP, suggesting that shorter exposure times or lower amounts of input protein would reveal two bands with anti-GFP. **(B)** Plots showing the fraction of SUMOylated Kv4.2 channels in control, SUMO+Ubc9, and anacardic acid treatment groups. Each symbol represents one independent experiment, and all data points were normalized by the mean value for the control treatment group. Asterisk, significant differences in the fraction of SUMOylated Kv4.2 channels among treatment groups. One-way analysis of variance (ANOVA) with Tukey’s *post hoc*, *F*_(2,17)_ = 3.68, *p* = 0.047; control, 1.0 ± 0.16; SUMO+Ubc9, 1.6 ± 0.23; anacardic acid, 1.1 ± 0.083.

SUMOylation can be increased in HEK cells by overexpressing SUMO and its conjugating enzyme Ubc9 (Dai et al., 2009; Parker et al., 2017). SUMOylation can be decreased by the application of anacardic acid, which inhibits protein SUMOylation by binding to the SUMO-activating enzyme (E1) and preventing the formation of the E1-SUMO intermediate (Fukuda et al., 2009). To determine if SUMOylation of Kv4.2g could be manipulated in our heterologous expression system, we transiently transfected HEK-Kv4.2g cells with mCherry or mCherry+SUMO+Ubc9 plasmid DNA. For the anacardic acid treatment group, the drug was bath applied (100 μM) for 1 h to HEK-Kv4.2g cells that had been transiently transfected with mCherry immediately before cell lysis. Kv4.2g SUMOylation was analyzed with IP followed by western blotting experiments (Figure 3). Blots were initially probed with anti-SUMO-2/3 and then stripped and re-probed with anti-GFP (Figure 3A). The fraction of SUMOylated Kv4.2g channels in a given experiment was determined by dividing the BSA-normalized optical density (OD) of the SUMO-2/3 band by the BSA-normalized OD of the GFP band, as described in “Materials and Methods” section. All data for control (mCherry), SUMO+Ubc9, and anacardic acid treatment groups were plotted in Figure 3B after normalizing each data point by the mean control value. A significant ~60% increase in mean Kv4.2g channel SUMOylation was observed in the SUMO+Ubc9 treatment group relative to control (Figure 3B). No significant difference in Kv4.2g channel SUMOylation was observed in cells treated with anacardic acid relative to control (Figure 3B). This might be due to a low level of baseline SUMOylation in the HEK-Kv4.2g cell line. On the other hand, a higher concentration and/or a longer application of anacardic acid might be necessary to observe a decrease in Kv4.2g channel SUMOylation in our heterologous expression system. In sum, overexpressing SUMO+Ubc9 in HEK-Kv4.2g cells produces a significant increase in Kv4.2g SUMOylation, and only this manipulation was performed in subsequent experiments.

### Increased SUMOylation of Kv4.2 Channels Alters the Properties of I_A_

**Figure 4:**
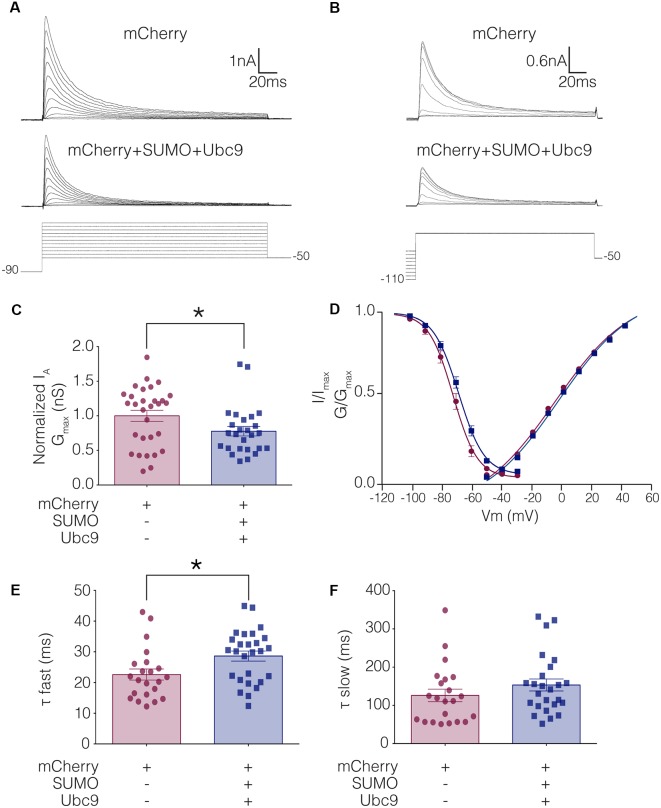
I_A_ G_max_ is significantly decreased in HEK-Kv4.2g cells transiently transfected with SUMO+Ubc9 compared to control. Whole cell patch clamp experiments were performed on HEK-Kv4.2g cells that were or were not transiently transfected with SUMO+Ubc9 to produce a ~60% increase in mean Kv4.2 SUMOylation. **(A)** Representative current traces (upper panel) and voltage steps (lower panel) used to obtain the voltage dependence of activation. **(B)** Representative current traces (upper panel) and voltage steps (lower panel) used to obtain the voltage dependence of steady-state inactivation. **(C)** Plots of normalized I_A_ G_max_ for control and SUMO+Ubc9 treatment groups. Each data point represents one cell. Cells in each treatment groups were obtained from ≥3 independent transfections. All data points were normalized by the mean value for the control treatment group. Asterisk, significantly different; control, 1.0 ± 0.079; SUMO+Ubc9, 0.78 ± 0.068; Mann-Whitney *U p* = 0.044. **(D)** Activation and steady-state inactivation curves for control and SUMO+Ubc9 treatment groups. Each point represents the mean ± SEM for all cells shown in panel **(C)**. **(E,F)** Inactivation τ for all cells shown in panel **(C)**. Asterisk, significantly different; *t*-test *p* = 0.018.

To test whether altering baseline SUMOylation affected the function of Kv4.2 channels, HEK-Kv4.2g cells were transiently transfected with mCherry (control) or mCherry+SUMO+Ubc9 plasmid DNA, and whole cell patch clamp recording was used to elicit I_A_ (Figure 4A) and steady-state inactivation of I_A_ (Figure 4B). The maximal conductance (G_max_; Figure 4C), voltage dependence (Figure 4D), and time constants (τ) of inactivation (Figures 4E,F) in the two treatment groups were measured. Enhancing baseline SUMOylation by ~60% (Figure 3) produced a significant ~22% decrease in mean I_A_ G_max_ (Figure 4C; Table 3), a small but significant ~4 mV depolarizing shift in the voltage-dependence of inactivation (Figure 4D, Table 3), and a significant ~25% increase in the fast time constant of inactivation (τ_f_; Figure 4E; Table 3).

**Table 3:** Whole-cell patch clamp physiology data for stable lines.

	Stable Lines							
	HEK-Kv4.2g		HEK-Kv4.2g K437R		HEK-Kv4.2g K579R		HEK-Kv4.2g K437R+K579R	
	mCherry	mCherry+SUMO+Ubc9	mCherry	mCherry+SUMO+Ubc9	mCherry	mCherry+SUMO+Ubc9	mCherry	mCherry+SUMO+Ubc9
G_max_ (nS)	32.7 ± 2.6	25.5 ± 2.2^T^	75.2 ± 6.0	49.5 ± 9.2^TT^	26.2 ± 4.3	26.7 ± 3.0^NS^	92.8 ± 13.3	84.5 ± 10.9^NS^
V_50_ Activation (mV)	−4.9 ± 2.0	−2.8 ± 1.7	−2.5 ± 1.9	−5.1 ± 1.6	−6.5 ± 1.5	−4.3 ± 2.6	−19.3 ± 3.8	−15.2 ± 4.3
V_50_ Inactivation (mV)	−72.2 ± 1.2	−67.8 ± 0.7^T^	−73.5 ± 1.1	−75.8 ± 1.7^NS^	−72.8 ± 1.0	−71.2 ± 1.1^NS^	−69.3 ± 1.6	−69.4 ± 1.9^NS^
Slope Activation	29.0 ± 1.3	25.6 ± 1.1	31.1 ± 1.9	29.8 ± 1.8	25.0 ± 1.3	26.4 ± 1.0	26.1 ± 2.0	29.3 ± 2.2
Slope Inactivation	−7.3 ± 0.3	−7.4 ± 0.3	−6.7 ± 0.2	−6.7 ± 0.2	−7.8 ± 0.3	−6.7 ± 0.2	−6.5 ± 0.4	−6.7 ± 0.3
τ_Fast_ (ms)	22.6 ± 1.8	28.6 ± 1.6^T^	16.6 ± 0.9	18.3 ± 2.0^NS^	29.4 ± 1.8	23.2 ± 1.7^TTT^	26.8 ± 2.8	18.3 ± 2.9^NS^
τ_Slow_ (ms)	126.1 ± 16.3	153.4 ± 15.7	64.1 ± 3.0	72.0 ± 8.9	229.8 ± 29.3	121.4 ± 12.9	81.04 ± 7.2	77.9 ± 10.9

*^T^HEK-Kv4.2g mCherry significantly different from mCherry+SUMO+Ubc9 (G_max_, Mann-Whitney U, *p* = 0.044; V_50_ inactivation, t-test, *p* = 0.0019; τ_fast_, t-test, *p* = 0.018). ^TT^HEK-Kv4.2g K437R mCherry significantly different from mCherry+SUMO+Ubc9 (t-test, *p* = 0.035). ^TTT^HEK-Kv4.2g K579R mCherry significantly different from mCherry+SUMO+Ubc9 (t-test, *p* = 0.019). ^NS^indicates not significant*.

### Kv4.2 SUMOylation Regulates Channel Surface Expression

**Figure 5:**
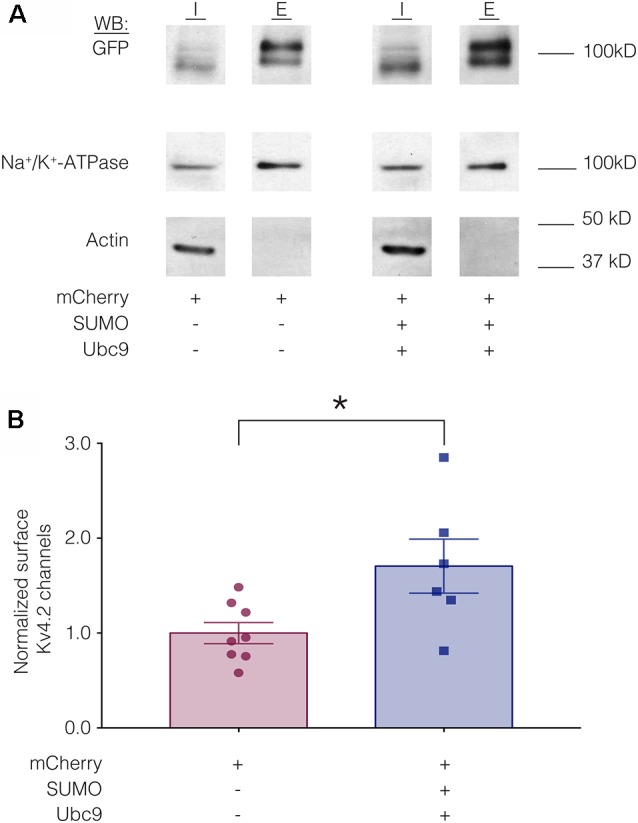
Increased SUMOylation mediates an increase in Kv4.2 surface expression. **(A)** Representative western blots for each treatment group. HEK-Kv4.2g cells were transiently transfected with mCherry or mCherry+SUMO+Ubc9. Extracellular proteins were biotinylated, and cells were lysed. Surface proteins were isolated using NeutrAvidin beads (E for extracellular). Unbound proteins were assumed to be intracellular (I for intracellular). The upper portion (above 50 kD) of a western blot containing I and E fractions was probed for GFP and then stripped and re-probed for Na^+^/K^+^-ATPase, and the lower portion of the blot (below 50 kD) was probed for actin. Intracellular bands appeared to run slightly lower than extracellular. There are several potential explanations including distinct loading buffers, different protein concentrations and differences in post-translational modifications. **(B)** Plots of normalized Kv4.2g channel surface expression. The blots were used to measure channel surface expression as described in “Materials and Methods” section. Each data point is one independent experiment. Each data point was normalized by the mean value for the control treatment group. Asterisk, significantly different; control, 1.0 ± 0.11; SUMO+Ubc9, 1.7 ± 0.28; *t*-test *p* = 0.025.

We hypothesized that a mean ~60% increase in Kv4.2 SUMOylation (Figure 3) produced a mean 22% decrease in I_A_ G_max_ (Figure 4) by reducing channel surface expression. To test this, biotinylation experiments were used to measure Kv4.2g channel surface expression in HEK-Kv4.2g cells transiently transfected with mCherry or mCherry+SUMO+Ubc9 plasmid DNA. Briefly, biotin was added to the cells to label extracellular proteins, cells were lysed, and biotinylated proteins were isolated using NeutrAvidin beads. Western blots containing intracellular and extracellular fractions were probed for GFP, Na^+^/K^+^-ATPase and actin as described in “Materials and Methods” section. We previously demonstrated that surface expression of Na^+^/K^+^-ATPase was not altered by increasing SUMO and Ubc9 (Parker et al., 2017), so changes in Kv4.2g surface expression between the two treatment groups were detected as differences in the ratio of extracellular GFP to Na^+^/K^+^-ATPase signals. A representative experiment is shown in Figure 5A. As expected, Kv4.2g and Na^+^/K^+^-ATPase were observed in intracellular and extracellular fractions, while actin was not. Extracellular Kv4.2g bands appeared higher than intracellular Kv4.2g bands. This could be due to differences in the loading buffers: Intracellular samples were run in 8M Urea loading buffer (8M urea, 20 mM Tris-HCl pH 8.0, 1 mM EDTA, 0.05% Bromophenol blue, 0.6M DTT), while extracellular samples were run in 1X SDS buffer (1X SDS, 0.1% Bromophenol blue, 100 mM DTT). Alternatively, protein concentrations and post-translational modifications could differ between fractions. Experiments were repeated and the extracellular GFP to Na^+^/K^+^-ATPase ratio was obtained for each experiment as described in “Materials and Methods” section. All data for control (mCherry) and experimental (mCherry+SUMO+Ubc9) treatments were plotted after normalizing each data point by the mean control value (Figure 5B). Surprisingly, enhancing Kv4.2g SUMOylation did not decrease channel surface expression as predicted, but rather, significantly increased Kv4.2g surface expression by ~70%.

### SUMOylation at K579 Is Responsible for the Decrease in I_A_ G_max_, While SUMOylation at K437 Mediates the Increase in Kv4.2 Surface Expression When SUMOylation Is Enhanced

**Figure 6:**
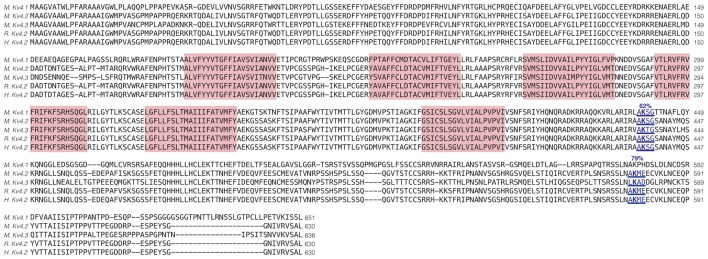
Identification of Kv4.2 SUMOylation sites. The amino acid sequences for mouse Kv4.1 (NP_032449.1), Kv4.2 (NP_062671.1), Kv4.3 (NP_001034436), rat Kv4.2 (Q63881.1), and human Kv4.2 (NP_036413.1) channels were aligned. Two putative, evolutionarily conserved SUMOylation sites predicted by SUMOplot and GPS-SUMO are located on intracellular domains. The probability that the site is SUMOylated is indicated, and transmembrane domains are highlighted in red.

Enhanced SUMOylation appears to have two opposing effects: (1) to decrease I_A_ G_max_ (Figure 4); and (2) to increase channel surface expression (Figure 5). One explanation may be that Kv4.2g can be SUMOylated at multiple sites, and SUMOylation at each site could produce a distinct effect. Potential Kv4 SUMOylation sites were identified using two free web-based prediction programs: SUMOplot^1^ and GPS-SUMO (Zhao et al., 2014). Multiple sites were identified, but only two were conserved across species and isoforms, were predicted by both programs and were located within intracellular domains (Figure 6). We chose to examine SUMOylation at these two lysine (K) residues, K437 and K579.

**Figure 7:**
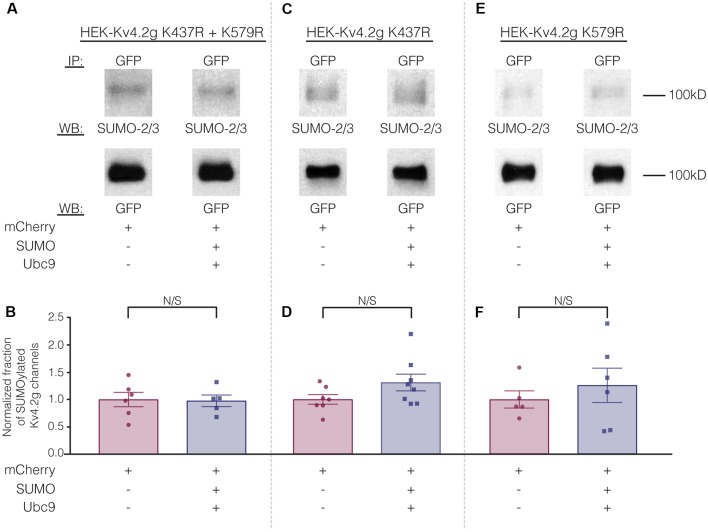
The ability to manipulate Kv4.2 channel SUMOylation is lost when K437 and K579 are mutated to R. **(A,C,E)** Representative experiments using the indicated stable mutant cell line. **(B,D,F)** Bar graphs showing normalized fraction of SUMOylated Kv4.2 channels. Each data point represents one independent experiment. All data points were normalized by the mean value for the mCherry treatment group. N/S indicates not significant. Kv4.2g K437R+K579R: control, 1.0 ± 0.13; SUMO+Ubc9, 0.98 ± 0.11; *t*-test *p* = 0.90. Kv4.2g K437R: control, 1.0 ± 0.088; SUMO+Ubc9, 1.3 ± 0.15; *t*-test *p* = 0.11. Kv4.2g K579R: control, 1.0 ± 0.16; SUMO+Ubc9, 1.3 ± 0.31; *t*-test *p* = 0.51.

To test whether overexpression of SUMO+Ubc9 increased SUMOylation at K437 and K579, we used site-directed mutagenesis to replace K with arginine (R). This mutation will prevent SUMOylation but not electrostatic interactions. Three stable lines were generated: HEK-Kv4.2g K437R+K579R, HEK-Kv4.2g K437R, and HEK-Kv4.2g K579R. SUMOylation was or was not globally enhanced in each line using transient transfection of mCherry+SUMO+Ubc9 or mCherry alone. SUMOylation of the Kv4.2 double mutant was highly similar in the two treatment groups (Figures 7A,B). On the other hand, average Kv4.2g SUMOylation was increased in the single mutants, but instead of the significant ~60% increase observed for wild-type channels (Figure 3), each single mutant showed a ~30% increase in SUMOylation that was not significantly different from control (Figures 7C–F). Together these data suggested that both sites were being SUMOylated and each contributed equally to the ~60% increase observed for wild-type Kv4.2g. It should be noted that Kv4.2g channels were SUMOylated in the double mutant, suggesting that additional sites on the channel or the GFP tag were SUMOylated under baseline conditions and that SUMOylation at these sites was not obviously increased by transient overexpression of SUMO+Ubc9.

**Figure 8:**
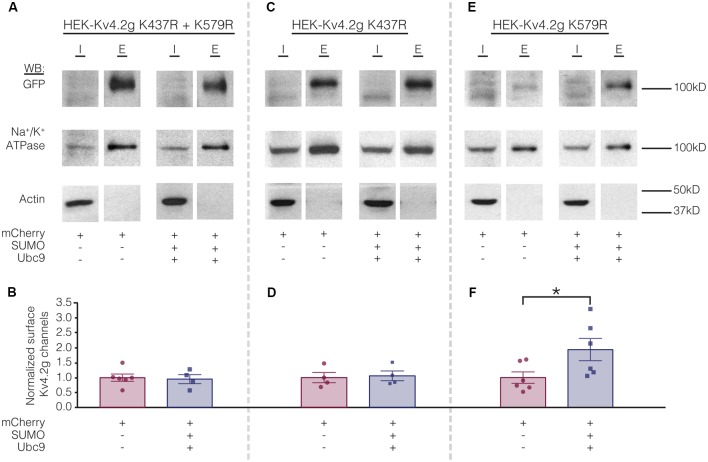
Increased SUMOylation at K437 mediates the increase in Kv4.2 surface expression. **(A,C,E)** Representative experiments. **(B,D,F)** Bar graphs showing normalized Kv4.2 surface expression. Each data point is one independent experiment. All data points were normalized by the mean value for the mCherry treatment group. Asterisk, significant difference. Kv4.2g K437R+K579R: control, 1.0 ± 0.12; SUMO+Ubc9, 0.95 ± 0.15; *t*-test *p* = 0.82. Kv4.2g K437R: control, 1.0 ± 0.17; SUMO+Ubc9, 1.1 ± 0.16; *t*-test *p* = 0.81. Kv4.2g K579R: control, 1.0 ± 0.19; SUMO+Ubc9, 1.9 ± 0.37; *t*-test *p* = 0.049.

Biotinylation experiments were used to measure channel surface expression in mutant cell lines transiently transfected with mCherry or mCherry+SUMO+Ubc9. Whereas overexpressing SUMO+Ubc9 in the wild-type cell line produced a significant 70% increase in Kv4.2g surface expression (Figure 5), globally enhancing SUMOylation in the double mutant or the HEK-Kv4.2g K437R cell lines had no effect on Kv4.2g surface expression (Figures 8A–D). In contrast, overexpressing SUMO+Ubc9 produced a significant 94% increase in Kv4.2g surface expression in HEK-Kv4.2g K579R cells relative to mCherry controls (Figures 8E,F). A *t*-test indicated that the 94% increase in the K579R mutant cell line was not significantly greater than the 70% increase observed in the wild-type cell line (*t*-test, *p* = 0.64).

**Figure 9:**
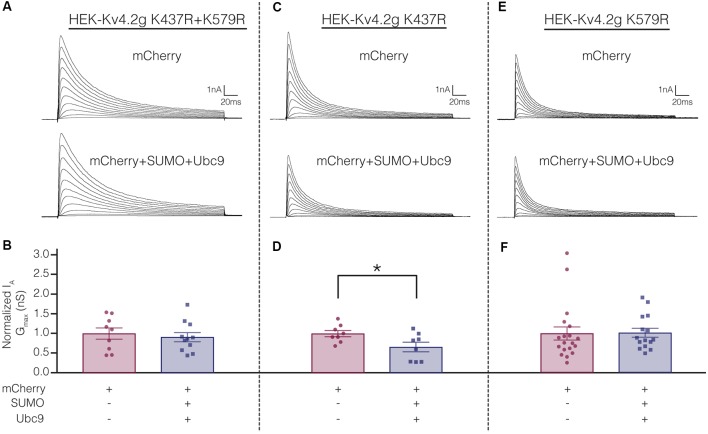
Increased SUMOylation at K579 mediates the decrease in I_A_ G_max_.** (A,C,E)** Representative current traces for the indicated stable mutant cell line in each treatment group. **(B,D,F)** Bar graphs representing normalized I_A_ G_max_. Each data point is one cell, and cells in each treatment group were obtained from ≥3 independent transfections. All data points were normalized by the mean value for the mCherry treatment group. Asterisk, significantly different. Kv4.2g K437R+K579R: control, 1.0 ± 0.14; SUMO+Ubc9, 0.91 ± 0.12;* t*-test *p* = 0.63. Kv4.2g K437R: control, 1.0 ± 0.080; SUMO+Ubc9, 0.66 ± 0.12; *t*-test *p* = 0.035. Kv4.2g K579R: control, 1.0 ± 0.1656; SUMO+Ubc9, 1.02 ± 0.1141; Mann-Whitney *U* *p* = 0.4715.

Electrophysiological experiments were repeated on the mutant cell lines to determine if SUMOylation at K437 and/or K579 was associated with the SUMOylation-mediated changes in Kv4.2g biophysical properties (Figure 4). Whole cell patch clamp was used to measure I_A_ in mutant cells lines transiently transfected with mCherry or mCherry+SUMO+Ubc9 (Figure 9; Table 3). Whereas over expressing SUMO+Ubc9 in wild-type HEK-Kv4.2g produced a significant 22% decrease in I_A_ G_max_ relative to mCherry controls (Figure 4B), enhancing SUMOylation did not reduce I_A_ G_max_ in the double mutant or the HEK-Kv4.2g K579R mutant cell lines; however, a significant decrease was observed in the HEK-Kv4.2g K437R cell line (Figure 9). These data suggested that SUMOylation of Kv4.2g at K579 mediates a decrease in I_A_ G_max_.

Table 3 shows that the SUMOylation-mediated 4 mV shift in the V_50_ inactivation seen in the wild-type channel (Figure 4C) was lost in cells expressing Kv4.2g channels containing the double-mutation or either of the single-mutations. Similarly, the previously observed SUMO-mediated increase in τ_f_ (Figure 4D) was lost in all three mutant cell lines, and a mean ~20% decrease was observed in the double mutant and K579R cell lines. Interpretation of these data is confounded by the fact that the mutations themselves may alter τ_f_ independent of SUMOylation, and/or there could be cell-line specific factors contributing to the effects. The latter hypothesis was next tested using transient transfection experiments (see “There Is a Significant Decrease in I_A_ G_max_ in HEK Cells Transiently Transfected With Kv4.2g K579R Compared to Kv4.2g” section below).

In sum, enhanced SUMOylation at K437 increased Kv4.2 channel surface expression while enhanced SUMOylation at K579 decreased I_A_ G_max_ (Figures 8, 9). Enhancing SUMOylation of the wild type channel produced a ~30% increase in SUMOylation at both sites (7), and the net result of the two opposing effects was a ~22% decrease in I_A_ G_max_ (Figure 4B). Interestingly, when the SUMOylation-mediated increase in surface expression was prevented by the K437R mutation, enhanced SUMOylation at K579 produced only a slightly larger decrease in the mean I_A_ G_max_ (33% instead of 22%; Table 3). Similarly, when the SUMOylation-mediated decrease in I_A_ G_max_ was prevented by the K579R mutation, enhancing SUMOylation at K437 produced an average 94% increase in surface expression (Figures 8E,F), but no change in I_A_ G_max_ (Figures 9E,F; Table 3). These data suggest that the channels inserted into the membrane upon enhanced SUMOylation may be mostly silent.

### There Is a Significant Decrease in I_A_ G_max_ in HEK Cells Transiently Transfected With Kv4.2g K579R Compared to Kv4.2g

**Figure 10:**
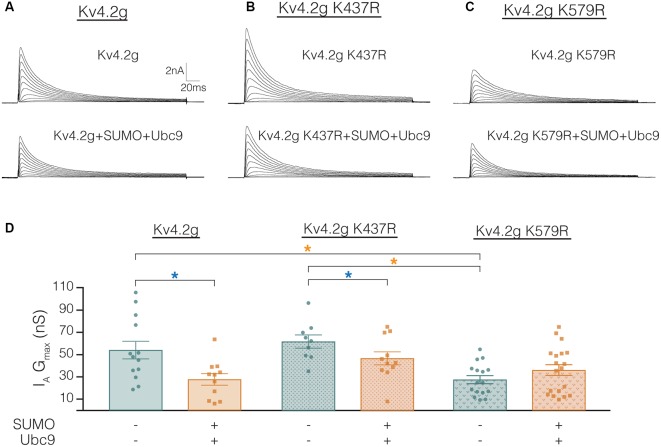
Transiently transfecting HEK cells with Kv4.2g K579R significantly decreases I_A_ G_max_ compared to control. **(A–C)** Representative current traces for each treatment group. **(D)** Plots showing I_A_ G_max_. Each data point represents one cell, and data for each treatment group were pooled from ≥3 independent transfections. Orange asterisks, significant differences in I_A_ G_max_ among HEK cells expressing Kv4.2g, Kv4.2g K437R, and Kv4.2g K579R. One-way ANOVA with Tukey’s *post hoc*, *F*_(2,35)_ = 9.75, *p* = 0.0004. Blue asterisks, significant differences in I_A_ when SUMO+Ubc9 was co-transfected. Kv4.2g: control, 54.2 nS ± 7.9; SUMO+Ubc9, 27.8 nS ± 5.2; *t*-test *p* = 0.014. Kv4.2g K437R: control, 62.1 nS ± 5.9; SUMO+Ubc9, 46.9 nS ± 5.9; *t*-test *p* = 0.049. Kv4.2g K579R: control, 27.6 nS ± 3.59; SUMO+Ubc9, 36.2 nS ± 4.84; *t*-test *p* = 0.18.

The mutations K437R and K579R prevented SUMOylation at these sites and the SUMOylation-mediated alterations in surface expression and I_A_ G_max_, respectively (Figures 8, 9). It was not clear if the mutations blocked, or mimicked and occluded the effects of SUMOylation. Measurements could not be compared between the independently selected stable lines because there could be differences in their proteomes and/or Kv4.2g gene copy number; i.e., different numbers of plasmids stably integrated into the genome in each cell line. In order to address this issue, HEK cells were transiently transfected with wild-type or mutant plasmids, and I_A_ was recorded using whole-cell patch clamp. Figure 10 illustrates that HEK cells transiently transfected with the K579R plasmid had a significantly smaller I_A_ G_max_ than cells transfected with the wild-type or K437R plasmids, while I_A_ in the latter two transfections was not significantly different (Kv4.2g, 54.2 nS ± 7.9; Kv4.2g K437R, 62.1 nS ± 5.9; Kv4.2g K579R, 27.6 nS ± 3.6). I_A_ could not be further reduced by enhancing SUMOylation in cells transfected with K579R plasmid, whereas this treatment did reduce I_A_ in cells transfected with the wild-type or K437R plasmids (Figure 10D). These data suggest that enhancing SUMOylation at K579 had the same effect as removing K579; i.e., the K579R mutation mimicked and occluded the effect of SUMOylation at K579.

Overexpression of SUMO+Ubc9 in the stable HEK-Kv4.2g cell line significantly shifted the V_50_ inact and increased τ_f_ (Figures 4D,E; Table 3). It is noteworthy that these significant changes were not observed in transient co-transfections with Kv4.2g+SUMO+Ubc9 (Table 4). Also, these parameters were not significantly different among transient transfections using wild-type vs. mutant plasmid DNA (Table 4). These data suggest that SUMOylation at K437 or K579 is not sufficient to regulate the V_50_ inact and τ_f_. The SUMOylation-mediated changes in the stable HEK-Kv4.2g cell line may have required an additional factor(s) specific to that cell line.

**Table 4:** Whole-cell patch clamp physiology data for transient transfections.

	Transient transfections					
	Kv4.2g		Kv4.2g K437R		Kv4.2g K579R	
	Kv4.2g	Kv4.2g+SUMO+Ubc9	Kv4.2g K437R	Kv4.2g K437R+SUMO+Ubc9	Kv4.2g K579R	Kv4.2g K579R+SUMO+Ubc9
G_max_ (nS)	54.2 ± 7.9^TTT^	27.8 ± 5.2^T^	62.1 ± 5.9^TTT^	46.9 ± 5.9^TT^	27.6 ± 3.6	36.2 ± 4.8
V_50_ Activation (mV)	−2.6 ± 1.8	2.5 ± 2.5	−5.1 ± 3.3	−0.7 ± 2.1	2.6 ± 1.8	−4.3 ± 3.4
V_50_ Inactivation (mV)	−71.5 ± 1.5	−72.9 ± 1.0^NS^	−69.6 ± 1.7	−70.8 ± 1.3	−73.5 ± 0.9	−70.9 ± 0.8
Slope Activation	26.1 ± 0.8	23.0 ± 1.0	27.1 ± 1.8	28.2 ± 1.4	27.2 ± 0.8	28.7 ± 1.1
Slope Inactivation	−6.4 ± 0.2	−7.0 ± 0.2	−6.2 ± 0.2	−6.3 ± 0.2	−7.1 ± 0.2	−6.5 ± 0.2
τ_Fast_ (ms)	22.1 ± 2.4	27.4 ± 3.1^NS^	17.1 ± 1.7	17.1 ± 1.0	26.1 ± 2.1	26.9 ± 2.0
τ_Slow_ (ms)	76.2 ± 8.0	105 ± 20.5	68.3 ± 6.9	60.9 ± 3.7	93.2 ± 10.1	138.4 ± 32.4

*^T^Kv4.2g significantly different from Kv4.2g+SUMO+Ubc9 (t-test, p = 0.014). ^TT^Kv4.2g K437R significantly different from Kv4.2g K437R+SUMO+Ubc9 (t-test, p = 0.049). ^TTT^Significantly different from Kv4.2g K579R (One-way ANOVA with Tukey’s post hoc, F_(2,35)_ = 9.75, p = 0.0004). ^NS^Significant differences produced by enhanced SUMOylation previously observed in the Kv4.2g stable line are not observed when Kv4.2g is transiently expressed (V_50_ inactivation, t-test p = 0.48; τ_Fast_, t-test p = 0.19)*.

**Figure 11:**
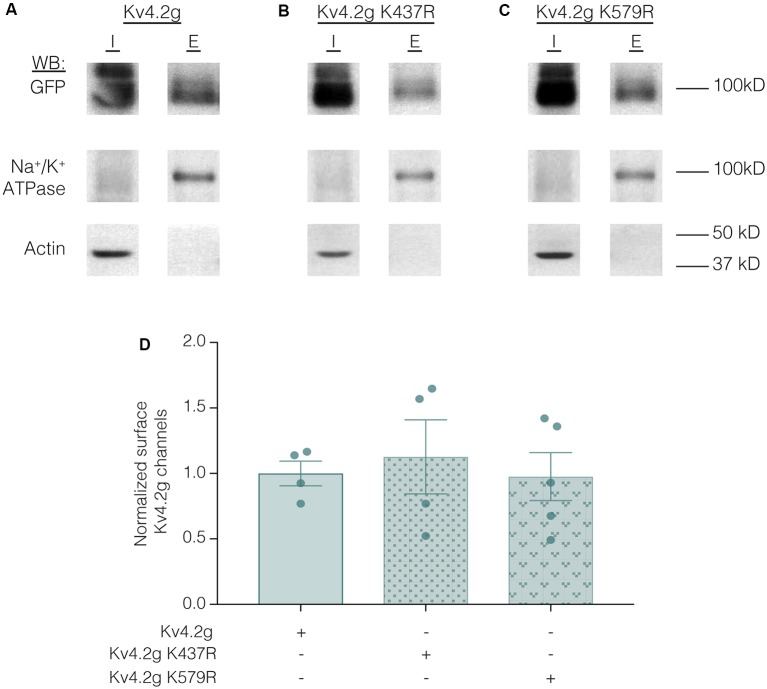
Transiently transfecting HEK cells with wild-type or mutant Kv4.2g does not significantly alter surface expression. HEK cells were transiently transfected with Kv4.2g, Kv4.2g K437R, or Kv4.2g K579R plasmid DNA and cell surface biotinylation experiments were performed. **(A–C)** Representative western blots for each treatment group. **(D)** Plots of normalized surface Kv4.2g channels. Each data point is one independent experiment and was normalized by the mean wild-type value. One-way ANOVA with Tukey’s *post hoc*, *F*_(2,10)_ = 0.16, *p* = 0.85.

Lastly, we examined whether the K437R mutation blocked, or mimicked and occluded the effect of K437 SUMOylation. HEK cells were transiently transfected with wild-type or mutant plasmids, and biotinylation experiments were performed. Figure 11 illustrates that Kv4.2g surface expression was not significantly different among HEK cells transiently transfected with wild-type or mutant plasmids (Kv4.2g 1.0 ± 0.094, Kv4.2g K437R 1.1 ± 0.28, Kv4.2g K579R 0.98 ± 0.18). Thus, the K437R mutation blocks the effect of SUMOylation.

## Discussion

Several ion channels are post-translationally regulated by SUMOylation (Kruse et al., 2009; Wasik and Filipek, 2014; Gong et al., 2016; Parker et al., 2017; Wu et al., 2016; Henley et al., 2018). Multiple members of the voltage-gated potassium channel superfamily are known to be SUMOylated including Kv1.1 (Qi et al., 2014), Kv1.5 (Benson et al., 2007), Kv2.1 (Dai et al., 2009; Plant et al., 2011), Kv7.1 (Xiong et al., 2017), Kv7.2 (Qi et al., 2014), and Kv11.1 (Steffensen et al., 2018). In this work, we tested the hypothesis that Kv4 channels can be SUMOylated to regulate their biophysical properties and surface expression. We found Kv4.2 channels were decorated by SUMO-2/3 but not SUMO-1 in rat membrane preparations. *In silico* analysis suggested that Kv4.2 could be SUMOylated at several sites. Two predicted SUMOylation sites, K437 and K579, were conserved across species and Kv4 isoforms. Globally enhancing baseline SUMOylation in HEK cells stably expressing Kv4.2 increased SUMOylation at K437 and K579 by ~30% each. Enhanced SUMOylation at K437 resulted in a mean ~70%–95% increase in Kv4.2 surface expression with no change in I_A_ G_max_. Enhancing SUMOylation at K579 decreased mean I_A_ G_max_ by ~25%–50% with no change in surface expression. Our data support the hypothesis that post-translational SUMOylation of Kv4 channels can regulate their function through multiple mechanisms.

### The Function of Kv4 Channel SUMOylation

Post-translational modification of Kv4 channels regulates I_A_ over short time scales. This is the first report that Kv4.2 channels can be post-translationally modified by SUMO. Globally increasing SUMOylation in HEK cells expressing a mouse Kv4.2-GFP fusion protein (Kv4.2g) increased Kv4.2 SUMOylation on the C-terminus at K437 and K579. When both sites were mutated to R, Kv4.2g SUMOylation could no longer be experimentally increased; however, Kv4.2 was still SUMOylated under baseline conditions. This suggests that SUMOylation occurs at additional sites on the channel and/or the GFP tag.

There are three potential, non-mutually exclusive consequences of protein SUMOylation. First, SUMOylation can prevent protein-protein interactions through steric hindrance (Dustrude et al., 2016). Second, SUMO can compete with other post-translational modifications that occur on the same K residue such as ubiquitination, methylation, or acetylation (Anderson et al., 2012; Wang et al., 2014). The third and most common function of SUMOylation is to promote protein-protein interactions (Psakhye and Jentsch, 2012; Seifert et al., 2015). SUMO interacts with specific protein binding domains, and the best studied is the SUMO-interacting motif (SIM; Hecker et al., 2006; Kerscher, 2007; Chang et al., 2011; Namanja et al., 2012; Aguilar-Martinez et al., 2015; Jardin et al., 2015). In general, the SUMO-SIM bond is relatively weak and serves to increase the affinity between proteins that bind one another through additional contacts. Thus, SUMOylation may stabilize an existing interaction and/or facilitate recruitment of a protein.

In some cases, SUMOylation enzymes can target an entire group of physically or functionally connected proteins. Importantly, a protein that is SUMOylated often possesses one or more SIMs. Thus, group SUMOylation can result in a set of interlocking Lego-like interactions for each protein in a complex, and the collection of individually weak SUMO-SIM bonds stabilizes the entire structure (Jentsch and Psakhye, 2013). In native cells, Kv4 channels exist in a ternary complex comprising four α-subunits, four cytoplasmic KChIP subunits, and four transmembrane DPPL subunits (Jerng et al., 2005; Amarillo et al., 2008; Foeger et al., 2012; Jerng and Pfaffinger, 2012; Wang et al., 2015). It is possible that group SUMOylation could stabilize the ternary complex, as prediction software indicates that all Kv4 channels, all KChIPs (1–4) and DPP6/10 possess multiple potential SUMO and SIM domains. In support of this idea, both KChIPs and DPPLs regulate Kv4 surface expression and gating (Jerng and Pfaffinger, 2014), and both have the potential to interact with Kv4 C-terminal SUMOylation sites (Sokolova et al., 2003; Callsen et al., 2005; Ren et al., 2005; Han et al., 2006; Lin et al., 2014). It is also possible that cytoplasmic C-terminal SUMOylation could regulate interactions between the Kv4.2 α-subunits, themselves, or C-terminal intra-molecular interactions (Hatano et al., 2004). SUMOylation could also regulate Kv4α interactions outside the ternary complex, as the C-terminus is known to interact with additional proteins, like SAP97 (Gardoni et al., 2007; El-Haou et al., 2009; Jerng and Pfaffinger, 2014).

In HEK cells stably or transiently expressing Kv4.2g, enhancing SUMOylation at K437 increased channel surface expression by up to 95% without altering I_A_ G_max_, suggesting SUMOylation increased the number of silent channels in the plasma membrane. The human protein atlas indicates KChIP2-4 are endogenously expressed at a low but detectable level in HEK cells^2^, and a previous study showed that co-expression of Kv4.2 and KChIP2 in HEK cells produced a 40-fold increase in Kv4.2 surface expression but only a 3-fold increase in Kv4.2 current density (Foeger et al., 2010). These data suggest SUMOylation at K437 could modulate the Kv4.2-KChIP interaction. SUMOylation could also stabilize interactions with endogenous SAP97 which is expressed at high levels in HEK cells^2^ and has been shown to increase Kv4 surface expression. On the other hand, attachment of a bulky SUMO at K437 might also reduce C-terminal interactions necessary for endocytosis (Nestor and Hoffman, 2012).

In HEK cells stably or transiently expressing Kv4.2g, increasing SUMOylation at K579 reduced I_A_ G_max_ by ~30%–50% without altering channel surface expression. Replacing K with R had the same effect. Since enhancing K579 SUMOylation produced the same effect as removing K579, SUMOylation most likely blocked an interaction involving K579 that influenced peak current amplitude.

### Physiological Functions and Regulation of SUMOylation

SUMO is emerging as an important physiological regulator of ionic currents. Dopamine gates activity-dependent SUMOylation to regulate the densities of I_A_ and the hyperpolarization-activated current (I_h_), and this is necessary to maintain activity homeostasis in a pattern generating neuron (Parker et al., 2019). In hippocampal neurons, SUMOylation is known to regulate the kainate-receptor-mediated excitatory postsynaptic current (Martin et al., 2007; Chamberlain et al., 2012), the delayed rectifier (Plant et al., 2011), and the M-current (Qi et al., 2014). SUMOylation is necessary for synaptic plasticity at mossy fiber-CA3 synapses, and hyper-SUMOylation suppresses the M-current and leads to hippocampal neuron hyperexcitability and seizures in a mouse model of sudden death. In dorsal root ganglion (DRG) sensory neurons, SUMOylation increases Na^+^ current (I_Na_) amplitude (Dustrude et al., 2016; François-Moutal et al., 2018; Moutal et al., 2018), and lowers the temperature threshold of activation for the TRPV1-mediated current (Wang et al., 2018) and hyper-SUMOylation contributes to pathological pain states. In rat cerebellar granule neurons, SUMOylation increases I_Na_ and reduces the leak current; and, hypoxia acts through SUMOylation to increase I_Na_, which contributes to hypoxic brain damage (Plant et al., 2012, 2016). In cardiac myocytes, SUMOylation determines the native attributes of the slow delayed rectifier current (I_Ks_; Xiong et al., 2017).

In many of the previous examples, SUMO-mediated regulation of the current was due to SUMOylation of ion channels and/or their auxiliary proteins. In hippocampal neurons, GluR2, Kv1.1, Kv2.1 and Kv7 ion channels are SUMOylated (Martin et al., 2007; Plant et al., 2011; Chamberlain et al., 2012; Qi et al., 2014). In DRG sensory neurons, auxiliary subunit Collapsin Response Mediator Protein 2 (CRMP2) is SUMOylated to increase its association with the voltage-gated sodium channel 1.7 (Na_V_1.7; Dustrude et al., 2013, 2016). Na_V_1.2 α-subunits and two-P domain, acid sensitive K^+^ (TASK) channels are SUMOylated in cerebellar granule neurons (Plant et al., 2012, 2016). In cardiac myocytes, KCNQ1 subunits are SUMOylated to shift their voltage dependence (Xiong et al., 2017). An appreciation for the regulation of ion channel SUMOylation is guiding the design of small molecules to prevent SUMO dysregulation in disease states (Cox and Huber, 2018; François-Moutal et al., 2018).

SUMOylation is highly regulated. In this study, target protein SUMOylation was experimentally enhanced by transient overexpression of the SUMO substrate and the SUMO conjugating enzyme, Ubc9. In native cells, SUMOylation can be regulated at the level of the target protein and/or the SUMOylation machinery. The target protein phosphorylation status gates its ability to be SUMOylated (Flotho and Melchior, 2013; Dustrude et al., 2016) and neuromodulators have been shown to act through kinases to gate SUMOylation (Parker et al., 2019). In addition, neuronal activity can regulate the SUMOylation machinery abundance, localization and level of activity (Lu et al., 2009; Craig et al., 2012; Hickey et al., 2012; Feligioni et al., 2013; Loriol et al., 2013, 2014; Lee et al., 2014; Nayak and Müller, 2014). Modulators and activity regulate I_A_ in native neurons (Kim et al., 2007; Lei et al., 2008; Shen et al., 2008; Shah et al., 2010; Rodgers et al., 2013; Parker et al., 2019). It will be important to test whether SUMO post-translational modification of Kv4.2 channels contributes to this regulation.

## Ethics Statement

This study was carried out in accordance with the principles of the Basel Declaration and recommendations of Ethical Issues of the International Association for the Study of Pain and National Institutes of Health. The protocol was approved by the Institutional Animal Care and Use Committee at Georgia State University.

## Author Contributions

MW and DB were responsible for the conception and design of the research presented. MW, LF, SA and DB contributed to the acquisition and analysis of the data. MW and DB were responsible for drafting the manuscript, and all authors were involved in revising the manuscript.

## Conflict of Interest Statement

The authors declare that the research was conducted in the absence of any commercial or financial relationships that could be construed as a potential conflict of interest.

## Footnotes

^1^http://www.abgent.com/sumoplot

^2^http://www.proteinatlas.org
